# Direct and indirect pathways of the basal ganglia: opponents or collaborators?

**DOI:** 10.3389/fnana.2015.00020

**Published:** 2015-03-02

**Authors:** Kainan S. Wang, John P. McClure, Samar K. Alselehdar, Vasiliki Kanta

**Affiliations:** Rutgers, The State University of New JerseyNewark, NJ, USA

**Keywords:** basal ganglia, medium spiny neuron, direct pathway, indirect pathway, striatum, locomotor activity

Imagine yourself walking down the street. The act of initiating the first step triggers an increased firing of some medium spiny neurons (MSNs) in the striatum. These MSNs are part of the direct pathway in the basal ganglia complex (for a review on the basal ganglia, see Parent and Hazrati, 1995), referred to as direct MSNs (dMSNs) henceforth. When you have finally reached your destination, different MSNs, which make up the indirect pathway (iMSNs), start to increase their firing so that you eventually come to a stop. In this way, the two groups of MSNs and their separate pathways have opposing effects on voluntary movement. Nevertheless, this dichotomous view of how the basal ganglia modulate locomotion is too simple to account for all the myriad of fine movements that animals can do. Indeed, current research making use of temporally- and spatially-precise techniques has called into question this canonical interpretation of the role of the two pathways.

In a recent publication in *Nature*, Cui et al. (2013) described a novel technique that enabled them to observe the activity of iMSNs and dMSNs in behaving animals. The researchers used *in-vivo* photometry to observe what is active in the striatum when mice are performing an operant task. The changes in fluorescence intensity of GCaMP3, a calcium indicator, correlated to the MSN activity. In particular, Cui et al. (2013) observed that both dMSNs and iMSNs increased their activity during “active states” of the task and remained quiescent when the mice were not moving. This finding alluded to concurrent activity of the two cell groups during motor initiation and motor suppression and cast doubt on the view of two functionally opposing pathways in the basal ganglia.

For every action, there is an equal and opposite reaction. The opposite reaction to the Cui et al. (2013) paper is a publication by Freeze et al. (2013) in *The Journal of Neuroscience*. In their experiment, Freeze et al. (2013) investigated the effects of optogenetically-activating dMSNs and iMSNs in freely moving animals. The authors selectively controlled the activation of either pathway by virally expressing channelrhodopsin-2 (ChR2) in Cre-expressing neurons. To determine the activation effects, they recorded neural activity within the basal ganglia output neurons in the substantia nigra pars reticulate (SNr) and monitored locomotion of the animals in an open field. The authors found that stimulating dMSNs predicted locomotor initiation whereas activation of iMSNs resulted in locomotor suppression.

Nevertheless, it is critical that we should approach the conclusions of the Freeze et al. (2013) paper with caution due to two experimental shortcomings. First, the investigators observed that activation of dMSNs or iMSNs both led to excited and inhibited subsets of SNr neurons. This finding suggested that activities within both pathways could influence SNr neurons in more than one way. This is in contrast to the canonical notion that dMSNs inhibit neuronal firing in the SNr and the iMSNs increase neuronal firing in the SNr. This finding also alluded to the idea that the two pathways are not segregated as previously postulated. Therefore, it is important to point out that optogenetically activating either the direct or indirect pathway does not faithfully portray how these pathways function under physiological conditions. Second, optogenetic activation of dMSNs and iMSNs removes the selectivity of cortical inputs into the striatum. It has been shown that cortical afferent projections into striatum are selective with some afferents (cortical intratelencephalically projecting type cells) synapsing onto dMSNs while other afferents (cortical pyramidal tract cells) synapse directly onto iMSNs (Reiner et al., 2010). This suggests that striatal output to the SNr neurons is dependent on specific cortical inputs, which was altogether disregarded in the use of optogenetic activation as described by Freeze et al. (2013).

In light of the results in Cui et al. (2013), it is postulated that the two pathways are not completely segregated as previously thought. This leads to three possible explanations describing the interactions between the two pathways and their respective cell groups. First, there could be a link between the two pathways in which dMSNs and iMSNs can influence each other's activity. The support for a link between the two pathways are strengthened in the Cazorla et al. (2014) reporting of “bridging collaterals” in the globus pallidus external segment (GPe), which were regulated in density by the excitability of iMSNs. When the dMSNs are activated, they, like their indirect pathway counterparts, also have the ability to massively inhibit the GPe cells via the bridging collaterals. This changes the canonical notion that the direct pathway is a monosynaptic transmission from the striatum to the SNr and suggests that upon chronic activation of the iMSNs, the dMSNs will recruit more bridging collaterals in the GPe to chime in on the inhibition of locomotor activity. Second, the concept of action selection is introduced (Mink, 1996), which postulates that the direct pathway functions to promote selection and initiation of a particular movement, while the indirect pathway neurons suppress competing, unwanted movements. In this model, a particular action initiation signal from the cortex requires both dMSNs and iMSNs cells to fine-tune the final output from the SNr cells. Third, Nadjar et al. (2006) challenged the view of segregated D1/D2 dopaminergic modulation on the two pathways by showing plasticity and co-expression of D1R and D2R in both types of MSNs. This experimental result suggests that the two populations of MSNs are not as anatomically distinct and lends strength to the dopamine-dependent synaptic plasticity model proposed by Calabresi et al. (2014).

Let us return to that walk down the street. When we make those first steps during action initiation, both dMSNs and iMSNs are concurrently active to help funnel the coarse cortical afferents through the basal ganglia. This type of pruning allows the dMSNs to help activate the right sets of muscles to move forward while permitting the iMSNs to help inhibit other muscles that can disrupt our balance or the forward motion. Ultimately, it is a coordinated effort between the two pathways that gets us to the final destination.

## Conflict of interest statement

The authors declare that the research was conducted in the absence of any commercial or financial relationships that could be construed as a potential conflict of interest.
